# Supplementary material for: Are carbon emissions trading and green financial instruments synergistic? -Comprehensive quantitative research based on content analysis
Source: PLoS One. 2024 Mar 7;19(3):e0298601. doi: 10.1371/journal.pone.0298601 (PMC10919601; doi:10.1371/journal.pone.0298601)
Supplement: S1 Data — (ZIP) [file pone.0298601.s001.zip › Data/地级市Z_F工作报告.zip!/╣π╬≈/╖└│╟╕█╩╨/└┤╫╘╣1⁄2╓┌║┼í╛┬φ┐╦╩2╛▌═°í┐_2011.docx]

各位代表：

现在，我代表市人民政府作工作报告，请各位代表连同《防城港市国民经济和社会发展第十二个五年规划纲要（草案）》一并审议，并请各位政协委员和列席人员提出意见。

“十一五”时期工作回顾

“十一五”时期，我们在自治区党委、政府和市委的坚强领导下，在市人大、政协的监督和支持下，团结带领全市各族人民，以科学发展观为统领，抢抓机遇，求是创新，扎实工作，奋力攻坚，成功应对国际金融危机、自然灾害等挑战，有效克服发展中出现的各种困难，胜利完成了我市“十一五”规划目标任务，为“十二五”时期实现更好更快发展奠定了坚实基础。

一、综合实力跨越提升，经济发展成就非凡

“十一五”时期，是我市发展质量最好、发展速度最快、城乡面貌变化最大、开放成果最丰硕、群众得实惠最多的五年，是综合实力跨越提升，经济发展取得非凡成就的五年。初步统计，2010年全市GDP达到 317亿元，是2005年的3.2倍，是全区唯一GDP年均增长超过20%的地级市，人均GDP达到3.7万元，位列广西第一；财政收入35.1亿元，是2005年的4.4倍，年均增长34%。全市经济社会12个主要指标22个分指标全部实现“翻一番”以上，15项指标提前一年以上达到“十一五”目标。据中国社科院评估，我市城市竞争力提升速度在全国294个地级以上城市中排名第一，被评为“2009年中国城市科学发展城镇化质量示范城市”、“2010年中国城市科学发展转变经济发展方式示范城市”。

二、北部湾规划深入实施，重大项目历史性突破

我们紧紧抓住广西北部湾经济区发展上升为国家战略的重大历史机遇，深化市情认识，强化规划对接，完善发展思路，坚持以港兴市、以工兴市、以区位优势兴市，落实新兴港口工业城市、重要门户城市和中国海洋文化名城的定位，发展大工业、大物流、大商贸、大旅游，构建钢铁能源基地、商贸物流基地、加工制造基地、产业转移承接基地和国际滨海旅游胜地，全力推进经济区发展规划深入实施。通过强化投资拉动，连续三年开展以“双百工程”为主要载体的项目建设主题年活动，项目建设实现历史性突破，是我市历史上项目数量最多、投资规模最大、带动效应最好的时期。五年来，实施重大项目600项，总投资规模达3000亿元，全社会固定资产投资累计完成942.6亿元，是“十五”时期的7.4倍，年均增长53.8%。搭建融资平台，采取土地融资、项目融资、BT等多种融资方式，累计储备土地1.3万亩，政府性融资贷款规模前所未有，到位资金达56.7亿元，是“十五”时期的26.5倍，有力带动城市建设，支撑产业发展。以千万吨级钢铁基地、648万千瓦核电、71万吨铜镍等三大“天字号工程”为代表的一批重大项目落户，钢铁基地具备全面开工建设条件，核电一期、40万吨铜冶炼配套项目开工建设。防城港电厂、大海粮油扩建、广西农垦精制糖、中一重工、上思华润水泥等重大项目先后投产。总投资近50亿元的沿海基础设施建设大会战全面告捷，白石牙水库、海港500千伏变电工程等20个重点项目建成运行。现代交通网络加快构建，沙企一级公路、防城南至企沙铁路支线建成，沿海铁路扩能改造工程防城港段、钦崇高速公路上思段等重要交通干线加快推进。能源、水利、通信等基础设施日趋完善，发展基础进一步夯实。

三、产业结构调整优化，工业主导地位凸显

经济转方式调结构步伐加快。五年来，三次产业比例由26:36:38调整为14:52:34，一产占GDP比重降至15%以下，工业比重升至45.2%，工业主导地位凸显，农业经济形态向工业经济形态转变迈出坚实一步。农业结构由传统种植业为主加快向农工商一体化模式转变。工业经济加速集约化转型，建筑业高速增长。第三产业提档升级，传统服务业加快向现代服务业转变。《人民日报》、《经济日报》等主流媒体对我市转变发展方式的举措和成效给予高度评价。

“以工兴市”战略得到全面贯彻落实，工业实现高速发展。五年来，全部工业总产值由119亿元增加到496亿元，年均增长33%，由全区第11位上升至第7位。临港产业加快集聚，逐步形成粮油加工、冶金、电力、制糖、磷化、林产林化、水泥建材等主导产业，成为全国最大的磷酸出口基地及重要的粮油加工基地。2010年粮油食品产业园产值达185亿元，大西南临港工业园产值突破100亿元。全市规模以上工业企业由76家增至163家。自治区A类产业园区和“广西强优工业企业”均从无到有增至3个。超百亿产值园区和强优企业数量居广西沿海三市之首。

四、海湾城市加快崛起，“有港无市”迅速改观

我们深化城市发展潜在优势的认识，对城市规划建设再审视、重定位，高起点、高标准完成了城市总体规划和各专项规划，确定了“三岛三湾一核心六组团”的城市发展新格局。着力彰显海湾城市特色，打造中国海洋文化名城，“海在城中，城在海中”的全海景生态海湾城市知名度、美誉度快速提升。

为尽快改变“有港无市”的局面，近三年来，以“四沿线、四片区、两景观、两改造”为重点，连续实施城建“百项工程”，出台政策，盘活土地，以政府融资为牵引，全面带动社会投资，掀起前所未有的建设热潮。市行政中心建成使用，“还岛于港”迈出关键一步。行政中心片区、桃花湾南部片区城市格局基本形成。北部湾大道、江山大道、金花茶大道等主干道建成通车。北部湾广场、仙人山公园等设施建成开放，青少年活动中心、科技图书馆、桃花湾体育馆等公共场馆项目顺利推进。市县两级污水处理厂、生活垃圾处理厂建成运行。中央商务片区主楼109层的CBD项目启动建设，阳光海岸等大型商住区加快建设，一批临湾海景楼盘拔地而起，西湾海域整治工程全面启动，城市景观中心建设步伐提速，人居环境大为改观。旧城改造成效显著，一批小街小巷改造完成，富裕路世界风情步行街等改造项目加快实施。城镇化率由40.6%提高到46%，城市建成区面积由28.7平方公里扩大到50平方公里，大城市主框架基本形成，“三年明显变化”目标已经实现。东兴、上思城区建设日新月异，企沙、江平等小城镇建设加快。城市管理水平不断提高，城乡清洁工程深入开展，港城面貌焕然一新。

五、港航建设实现飞跃，港口带动效应显著

坚持“以港兴市”战略，以建设国际枢纽大港为目标，科学调整港口布局，形成大中小港口协调发展的格局。五年来，港口基础设施建设累计投入86亿元，建成13个5万吨级以上深水大泊位及一批配套航道，其中20万吨级码头1个、15万吨级泊位4个，成为我国第一个能同时靠泊5艘海峡型船舶的枢纽大港。发展“水转水”业务，提高到发装卸效率，拓展储运交易功能，成为我国南方大宗干散货集散中心。开通海铁集装箱联运五定班列和东北亚、东南亚集装箱直达航线，内外贸航线和定期航班分别增至18条、27个，集装箱吞吐量由10.5万标箱增加到25.1万标箱，是广西沿海最大的集装箱港。港航服务体系进一步完善，广西首个电子口岸海运物流服务平台开通运行，口岸联检中心高效运转，港口物流服务全面提升。全市港口货物年吞吐量由2377万吨增加到7650万吨，增长2.2倍，占广西沿海港口货物吞吐量近7成。航运业发展迅猛，拥有航运企业27家，总运力达到51.3万吨，比“十五”增长6.9倍。

港口带动效应显著，吸引一批影响深远的重大临港工业项目落户，形成一批临港主导产业，有效带动物流园区和物流企业的发展。“以港引工、以工促港、港城互动”良性发展的联动效应迅速显现，新兴港口工业城市形成。

六、商贸旅游繁荣兴旺，第三产业提速发展

生产性服务业加快发展，生活性服务业欣欣向荣，商贸、旅游文化、房地产、金融等产业发展成为新亮点。三产累计实现增加值371.5亿元，是“十五”时期的2.4倍。商贸物流繁荣，完成物流总体规划，海产品、红木、建材等一批专业市场建成，东湾、冲仑等物流园区建设加快推进。国际滨海旅游胜地加快打造，完成旅游发展总体规划，深入实施旅游文化基础设施和景区景点建设大会战，十大重点景区加快建设，“十个一”旅游文化品牌加快形成，旅游文化产业蓬勃发展。十万大山国家森林公园和京岛风景名胜区成为4A景区，京族哈节、海上国际龙舟节、高山漂流等品牌打响，“梦幻北部湾”成功启航演出，《腾飞防城港》、《防城港是个好地方》等防城港之歌唱响，东兴被评为中国“长寿之乡”，新增三星级以上酒店17家，旅游接待能力大为提高，游客人数、旅游收入大幅增长。房地产投资由2005年的2.6亿元增加到2010年的85亿元，商品房累计销售面积是“十五”期间的8倍以上。金融组织体系日益完善，拥有银行、保险、证券、小额贷款公司等各种金融机构28家，开展跨境贸易人民币结算试点，金融服务能力大为提高。新兴服务业加快发展，2010年实现增加值45.6亿元，占GDP比重由6.2%提高到14.6%。居民消费结构不断升级，2010年社会消费品零售总额达到51亿元，是2005年的2.2倍。

七、农业基础更加稳固，县域经济发展壮大

在稳定粮食生产的基础上，着力推进农业产业结构调整和产业化经营，加快发展特色农业和休闲农业，农业综合生产能力进一步提高，农业总产值年均增长5.8%。加快实施增粮工程，大力推广良种良法新技术，粮食生产稳步增长。农业产业化加快发展，建成糖料蔗、速生丰产林、水产养殖等一批工业原料基地，4家企业成为自治区级农业产业化重点龙头企业。以“红金”系列为代表的特色优势农业形成规模，“红姑娘”红薯等形成品牌。农业产业结构优化，水产畜牧业占大农业的比重达58%。大规模开展农村基础设施建设，建成农村道路623公里，除险加固水库86座，建成“三面光”水渠241公里，新增、恢复及改善灌溉面积累计达119万亩。实施“六统强基”工程，新农村建设成效显著，建成一批以“农家乐”、“渔家乐”等为主的生态休闲观光农业示范村。

县域经济跨越发展，县域生产总值年均增长19%，财政收入年均增长33%。2010年港口区、防城区、东兴市财政收入均突破6亿元，上思县达到4.4亿元。港口区服务重大项目和城市建设，全力开展征地搬迁安置，发展园区经济，综合实力跃居全市之首。防城区商贸物流、现代农业、特色旅游快速发展，实现经济社会发展新突破。上思县围绕农林资源优势，制糖、林板林化、水泥建材及生态旅游等资源型产业加快发展。东兴市发挥沿边优势，以商贸、旅游、房地产等产业推动县域经济发展。各县（市、区）多次获得“广西经济发展十佳县（市、区）”等荣誉，实现“县县先进县县红”。

八、改革事业稳步推进，开放合作谱写新篇

国企改革扎实推进，东湾交通公司等国有企业改组整合；市与城区管理体制进一步理顺，建立了“市级为主导、区级为主体、街道为基础”的城市管理体制；集体林权改革基本完成；上思县财政直管改革试点顺利推进，科教文卫等领域的改革稳步实施。开放合作不断拓展，外贸进出口总额由2005年的8.5亿美元增加到2010年的25.6亿美元，年均增长24.7%，对外经济依存度53%。2010年引进区外境内资金120亿元，是2005年的10倍，五年累计实际利用外资4.3亿美元，年均增长30.7%。我市企业到境外设立分公司、办事处8家，境外投资额5830万美元。东兴国门广场改造工程、东兴边民互市贸易区、里火等一批口岸设施建设加快，通关效率大幅提升。东兴重点开发开放试验区纳入国家发展战略。与港澳台、东盟、日韩等多区域合作不断深化，与越南下龙、韩国永同郡等城市结为友好城市。成功举办中越边境（东兴—芒街）商贸旅游博览会、中越边民友好大联欢、“旅游大篷车”、“城市规划和建设成果展示大篷车”巡游宣传等重大活动，大批政要人士、重要客商、知名艺术家纷至沓来，影响力不断扩大，树立了重要门户城市的新形象。

九、民本防港理念彰显，社会事业成就斐然

围绕“民本防港、和谐崛起”的目标，狠抓民生工作，年均投入增长50%以上，民生改善前所未有，被评为“全国十大关爱民生城市”。居民收入水平不断提高，城镇居民人均可支配收入年均增长19.5%，农民人均纯收入年均增长15.7%，达到5600元，排全区第一。新型农村合作医疗保障参合率达98%，城镇居民医疗保险参保率达91%，防城区、东兴市新型农村养老保险试点工作扎实开展。解决6860名困难企业职工医疗参保问题，大幅度提高城乡居民福利保障标准。建成保障住房3499套，完成农村危旧房改造1292户。就业工作进一步加强，新增就业15万人，城镇登记失业率低于全区控制线1.3个百分点。创造“文明征地，和谐搬迁”的防城港模式，将失地失海农民纳入城镇职工养老保障体系。减轻教育负担2.6亿元，不让一名学生因家庭经济困难失学。发放农村低保金7742.5万元，受益对象182.4万人次;新建五保村56个，支出五保资金4708.9万元。构建孤儿全方位救助保障体系，建立弱势群体档案并落实帮扶措施。深入实施兴边富民基础设施建设大会战和整村推进扶贫工作，54.8万群众受益。解决27万人的饮水难问题。

科教文卫体等各项社会事业全面发展。第四轮科技创新计划通过自治区验收。教育“两基”工作通过国家验收，并得到巩固提高。引进100多名高级专业人才。京族“哈节”、独弦琴弹奏艺术被列入国家非物质文化遗产名录，村村通广播电视工程提前完成。市第一人民医院和中医院创“三甲”工作顺利推进；建成82个标准化农村卫生室。人口与计生工作创国优、省优扎实推进，人口自然增长率控制在11‰范围内。全民健身活动深入开展。依法管理和使用国土资源，有效保护耕地，确保发展用地用海。蝉联全国“双拥模范城”，人防工作获全国先进，国防动员成效显著。生态环境持续改善，完成造林4.6万公顷，森林覆盖率达56%，节能减排如期完成。完成第二次全国经济普查，开展第六次全国人口普查，统计服务经济调控的能力进一步增强。扶持人口较少民族发展卓有成效。口岸联检部门服务水平提升。建成国家级有色金属矿产品重点实验室。气象服务经济社会发展能力增强。市场监管、国资管理、边海防、外事侨务、宗教、档案、修志、妇女儿童、残疾人、红十字、防震减灾等各项工作取得新成效。安全生产连续五年获全区先进，突发公共事件应急体系逐步建立。“五五”普法验收达标，社会治安综合治理成效明显，打击走私、贩毒力度加大，调处工作深入开展，社会稳定工作进一步加强。全市社会和谐稳定，民族团结，边疆安宁，人民安居乐业。

十、政府建设全面加强，行政能力明显提高

加强民主法制，建设法治政府。坚持重大事项民主决策，出台《关于加强科学民主决策提高行政执行力的意见》，召开107次政府常务会，审议1128个议题，不断提高民主决策和科学决策水平。自 觉接受人大和政协监督，广泛听取社会各方面的意见和建议，人大代表建议、政协委员提案办复率达100%。出台社会保障、财政资金管理等规范性文件125个，完善行政审批、招投标、政府采购等40多项政策制度，完成行政复议269宗、行政诉讼821件，政府透明度和公信力进一步提高。

加强服务型政府建设。完成市县两级政府机构改革，市本级部门由35个减少到31个，成立企沙工管委、江山旅管委、金融办、征地办、港口办等机构，以适应发展需要。建立市县两级政务服务中心，积极推进政务公开和电子政务，开展绩效考评和“百人评议机关”活动，机关作风明显转变，行政效能明显提高。加强廉政建设，重点开展重大项目和领域的反腐败工作。建立项目投资评审制度，五年共核减资金14.5亿元；强化项目竣工审计，全面开展政府采购，严格资金使用管理，共节约资金1.6亿元。

加强行政能力建设。强化干部队伍教育培训，组织公务员培训565期7.1万人次，公务员思想观念不断转变。各级各部门驾驭发展能力、社会管理能力、公共服务能力进一步提高，妥善处理一批土地、债务等历史遗留问题，有效破解一系列发展瓶颈。投资发展环境不断优化，企业对我市投资环境满意度连续三年达到高度满意区间，荣获 “中国十佳和谐可持续发展城市”等称号。

各位代表！刚刚过去的2010年，面对金融危机余波未平、节能减排压力骤增等错综复杂的经济形势，我们坚持以科学发展观为统领，以“项目建设突破年”、“民生改善年”、“环境优化年”为主题，以“快增长、增投资、抓城建、上产业、拓开放、惠民生”为主攻方向，按照“谋划更早，布置更快，推进更实，效果更好”的工作要求，稳中求增抓一产，增中求快促二产，快中更快提三产，从年初就开始紧抓园区建设、“双百工程”、项目前期工作等事关经济发展全局的关键环节，及时采取有力的调控措施，牢牢把握工作主动权，有力推动了全市经济保持高速增长。初步统计,全年生产总值增长17%；财政收入增长28.2%；规模以上工业产值增长32.7%；全社会固定资产投资增长45.6%；外贸进出口总额增长18.1%；城镇登记失业率、人口自然增长率、万元地区生产总值能耗、化学需氧量排放总量、二氧化硫排放总量控制在计划以内。主要经济指标增幅或总量继续位居全区前列，较好地完成了市四届人大六次会议审议通过的目标任务。

各位代表！“十一五”的非凡成就来之不易，凝聚着全市人民的智慧和心血。借此机会，我代表市人民政府，向全体代表、政协委员，向全市各条战线的广大干部群众、离退休老同志，向驻我市解放军、武警官兵，向各民主党派、工商联、无党派人士、人民团体，向关心支持我市现代化建设的港澳台同胞、海外侨胞、国际友人和各界人士，表示衷心的感谢和崇高的敬意！

回顾“十一五”时期不平凡的历程，我们深深地体会到：一是必须坚持党的领导，不断解放思想。认真贯彻落实市委决策，以科学发展观为统领，坚决冲破各种旧观念、旧体制的束缚，放开思想想问题，放开手脚干事业，从更大区域、更高层面、更远前景谋划发展思路，深化改革、扩大开放，以非常思路、非常魄力、非常举措强力推进各项工作。二是必须突出重点，统筹兼顾。牢牢把握事关经济发展全局的关键环节，把主要精力放在上项目、强工业、抓城建、兴三产、促开放等重点方面。转变经济发展方式，既注重扩大总量，又注重提升质量；既注重当前利益，又注重长远发展；既注重经济发展，又注重民生改善，促进全市经济社会又好又快发展。三是必须提高效能，强化执行。团结各方力量，自觉接受人大、政协监督，充分调动发挥全市上下积极性，同心发力，只争朝夕、废寝忘食地干，“白加黑”、“五加二”地干，“人一之我十之、人十之我百之”地干；通过创新举措、完善制度、落实责任，深入开展效能建设，切实转变工作作风，强化政府执行力，确保工作不折不扣地落到实处。四是必须加强调控，破解瓶颈。牢牢把握工作主动权，应势而变，及时采取有力调控措施，抓早、抓紧、抓准、抓实，着力破解一批制约我市发展的关键问题，带动全局发展，推动经济持续高速增长。五是必须以人为本，关爱民生。始终把最广大人民的根本利益作为一切工作的出发点和落脚点，在经济发展的基础上更加注重保障和改善民生，更加注重公平正义，更加注重扶弱济困，扩大公共服务，完善社会管理，让人民群众共享改革发展成果。

在总结成绩的同时，我们也清醒地认识到，我市经济社会发展仍然面临不少困难和问题。主要是：经济总量不大，整体实力不强；城市经济不发达，辐射和吸纳能力较弱；人才、资金、土地等瓶颈制约因素依然存在；一些事关群众利益的热点、难点问题有待进一步解决；政府工作还存在薄弱环节。对这些困难和问题，我们将高度重视并采取有效措施努力加以解决。

“十二五”时期主要任务

“十二五”时期是我市科学发展、跨越发展的关键时期，尽管前进的道路还面临许多挑战，但机遇大于挑战。随着新一轮西部大开发和北部湾经济区建设的深入推进，中国—东盟自由贸易区建成，东兴重点开发开放试验区建设纳入国家发展战略，我市将进入工业化、城镇化、国际化迅速发展的新“黄金期”。为此，必须把握趋势，科学谋划，扎实工作，努力实现“十二五”经济社会发展新跨越。

根据市委四届十一次全会通过的《关于制定国民经济和社会发展第十二个五年规划的建议》，我市“十二五”国民经济和社会发展的指导思想是：高举中国特色社会主义伟大旗帜，以邓小平理论和“三个代表”重要思想为指导，深入贯彻落实科学发展观，围绕“富民强市、幸福和谐”的总体思路，以科学发展为主题，以加快转变经济发展方式为主线，坚持以港兴市、以工强市、以区位优势旺市，积极推进工业化、城镇化、信息化、市场化、国际化，深化改革开放，推动科技创新，保障和改善民生，维护社会和谐稳定，大力发展大工业、大物流、大商贸、大旅游，率先构筑沿海发展新高地、倾力打造中国东盟合作第一城，努力把防城港市建设成为面向东盟乃至更大区域的钢铁能源基地、商贸物流基地、加工制造基地、产业转移承接基地和国际滨海旅游胜地，成为广西北部湾经济区生态友好、开放度高、活力迸发的新兴港口工业城市、重要门户城市、中国海洋文化名城和独具魅力的创新创意之城、生态宜居之城、幸福和谐之城，为全面建成小康社会打下坚实基础。

“十二五”期间，我市经济社会发展主要目标是：全市地区生产总值年均增长17%左右，人均生产总值年均增长10%左右，财政收入年均增长22%左右，全社会固定资产投资年均增长30%左右，全部工业增加值年均增长16%左右，外贸进出口总额年均增长15%左右，实际利用外资年均增长20%左右，引进内资年均增长20%左右，港口货物吞吐量年均增长20%左右。实施国民收入倍增计划，城镇居民人均可支配收入年均增长14%左右，农村居民人均纯收入年均增长13%左右，城镇年均新增就业人数2.5万人左右，城镇登记失业率控制在4.5%以内，单位地区生产总值能耗、主要污染物排放量等约束性指标控制在自治区要求范围内。并提出争取目标是：到“十二五”期末，全市生产总值超千亿元，财政收入超百亿元，农村居民人均纯收入超万元。

根据上述指导思想和发展目标，《纲要（草案）》明确提出“十二五”期间经济和社会发展的任务及措施。

一、加快建设东兴试验区，打造中国东盟合作第一城

必须举全市之力建设东兴试验区这个新的“天字号工程”。以东兴市和“三岛三湾”一带为核心，充分利用两个市场、两种资源，实施边海联动战略，进一步解放思想，先行先试，努力建成西部地区开放度高、体制灵活、辐射力强的新型经济特区和国际区域经济合作新高地、沿边开发开放新一极。充分利用越南和东南亚地区农林、矿产等资源，积极发展进出口资源加工业，形成越南等东盟国家进口资源和商品集散中心。构建面向东盟的国际商贸物流基地、加工及装备制造基地和国际滨海旅游胜地，努力打造成为中国与越南乃至东盟开放合作先行区、西部地区开发开放引领区、睦邻安邻富邻示范区和区域性国际大通道。以此为引领，进一步深化与大西南、珠三角、港澳台、东盟等国内外的多区域、全方位、多领域合作，大力发展对外贸易，扩大招商引资，完善口岸设施，全面提升对外开放水平，打造中国东盟合作第一城。

二、坚持以工强市战略，构筑沿海发展新高地

坚定不移地把工业放在优先发展的地位，重点抓好企沙工业区、大西南临港工业园等六大产业园区建设，提升园区承载能力，强力打造钢铁精品、有色金属、粮油食品、能源、装备制造、修造船六大临港工业基地；发展壮大化工、建材、造纸与木材加工、农产品加工、医药制造等传统优势产业；加快发展海洋、新材料、新能源等新兴产业；实施企业集团战略，增强企业自主创新能力，培育一批大企业大集团。钢铁、核电、铜镍三大“天字号工程”建成投产。推动产业结构优化升级，形成一批销售收入超百亿元的产业。到2015年，全市工业增加值达到500亿元。

三、构建大城市格局，打造海湾宜居新城

以“造新城”为目标，深化“三岛三湾”布局，进一步完善城市规划，突出“海湾、门户、生态”城市特色，按照“双连东拓西延”的空间发展策略，以城市核心区、企沙半岛、江山半岛、江平新城为城市发展重点区域，拉开城市框架，构建“一核心六组团”的大城市格局。加快城市核心区建设，形成行政、金融、商务中心。加快渔氵万组团和西湾新城组团建设，建成集商务会议、文化创意、休闲娱乐、高端居住等为一体的国际生态新城。加快形成滨海、十万大山、沿边三大城镇带。适度超前配置医疗卫生、教育、水电、公共交通和商业设施等基本公共服务设施，不断完善城市功能。到2015年，中心城区人口达45万人，建成区面积80平方公里以上。

四、坚持以港兴市战略，建设国际枢纽大港

按照建设组合港发展方向，加快形成功能齐全、分类明确、吞吐量大、班期航线多、集疏运快捷、大中小泊位相匹配的现代化国际枢纽大港。到2015年，港口货物吞吐能力超过2亿吨，集装箱吞吐能力达270万标箱。合理开发利用岸线资源，加快港口基础设施建设。重点建设渔氵万港区、企沙港区、江山港区，统筹发展茅岭、竹山等中小港口群，构建“三区一群”港口发展新格局。完成渔氵万港区东岸线开发，形成环岛式港区布局，向南延伸规划建设深水大能力泊位，形成一批20-30万吨级专用公共泊位和40万吨级干散货码头，增强核心竞争力。企沙港区开工建设钢铁、铜镍等项目专用码头，形成以临海大型企业专用码头为主的新港区。江山港区重点建设国际客运中心和游艇基地，形成旅游客运港区。加快推进建设30万吨级三牙深水航道和15万吨级西湾航道等工程。建立和完善港口大通关体系和港航服务体系，完善铁路、公路集疏运系统，加强港口合作交流。

五、加强基础设施建设，提升发展支撑能力

按照“统筹规划、加快建设、适度超前、完善网络、健全体系、提高能力、适应发展”的原则，加快交通、水利、能源、信息化等基础设施建设，构建联通西南、中南、华南的陆海空交通网络，打造面向东盟的综合交通枢纽。公路方面，以高速公路为骨架，以一、二级公路为主干，着力构建出海出边大通道，完善与北部湾周边城市大通道网、区县间公路网和产业园区基础设施。加快建成防城至东兴高速公路、防城至峒中高等级公路、广西滨海公路防城港段。规划建设上思至企沙一级公路、企沙至茅岭一级公路等工程，进一步完善园区之间公路网络。铁路方面，加快建设沿海铁路扩能改造工程防城港段，规划建设防城至东兴高速铁路及其支线、防城港铁路集装箱办理站等铁路项目。开展防城港支线机场的前期工作。强化水利基础设施建设，启动建设大垌、大菉水库等供水工程，着力建设民生水利。加快推进灌区续建配套与节水改造步伐，不断提高抗旱能力。加强能源建设，加快建设核电、防城港电厂二期、抽水蓄能等重大电源项目，继续加强城乡电网和农村电网建设。加快覆盖城乡的信息基础设施建设。

六、统筹城乡一体化发展，建设社会主义新农村

推进“六个一体化”，打破城乡二元结构，实现城乡共同繁荣发展。重点发展钢铁、铜镍等配套产业，壮大园区经济，强力推进县域经济发展。加快小城镇发展步伐。强化城镇聚集与辐射带动功能，支持企沙、江平、那良、在妙等重点镇率先发展，积极发展茅岭、光坡、叫安、马路等资源较好的城镇。着力建立城乡统一的规划体系，加快推进城乡基础设施、产业、公共服务、市场体制等城乡经济社会一体化发展。积极发展现代农业，稳定粮食生产，调整种养结构，发展海洋渔业，积极培育“红金”等特色产业，加快推进海产品、香料香精、中草药等特色农产品生产加工基地建设，发展壮大龙头企业。坚持高起点、高标准、有特色原则，强化规划指导，建设一批新农村示范点，加快推进社会主义新农村建设。实施“六统强基”和“八到农家”工程，抓好城乡风貌改造，改善农村生产生活条件，推进生态文明富裕新农村建设。

七、构建商贸物流中心，打造国际滨海旅游胜地

大力发展以商贸、旅游、物流、金融、房地产为重点的现代服务业。以中央商务区为中心，不断完善全市商业网点，形成完备的商业网络体系，加快发展金融业，积极推动房地产业发展，充实提升生活性服务业。构建边境贸易产业组团，打造中国东盟区域性的国际商贸中心。大力发展以物流信息平台、综合物流园区、专业物流中心和配送中心为基础的现代物流服务网络，构建支撑本市产业发展、服务周边地区的现代物流中心，大力发展生产性服务业。积极促进跨国旅游便利化，形成面向越南等东盟国家的国际陆海旅游通道和“泛北部湾三小时国际旅游圈”，构建北部湾国际旅游目的地。打造十大重点旅游景区，建设以休闲度假、商务会展、长寿养生为主要功能的国际滨海旅游胜地。以“梦幻北部湾”带动发展一批文化精品，建设中国东盟文化交流合作基地，打造中国海洋文化名城。

八、深化体制机制改革，为发展提供强大动力

全面深化体制改革，在对外开放、财税金融、土地管理、人才开发等方面大胆探索，先行先试。切实转变政府职能，深化行政管理体制改革。深化国有体制、公共财政体制、投融资体制、价格体制等经济体制改革，发展壮大非公有制经济，健全市场体系。推进教育、科技、医药卫生、文化等社会事业领域改革。为经济社会快速、持续、协调发展提供强大动力。

九、坚持可持续发展，建设生态文明城市

加强节能减排工作，积极发展循环经济，构建资源循环利用产业链。加大生态保护力度，到2015年，全市森林覆盖率达到60%左右。开展环境综合防治，合理利用和保护资源，城镇污水集中处理率力争达到70%以上，城镇生活垃圾无害化处理率达到90%以上，集中式饮用水源水质100%达标。积极开发和集约节约利用土地，切实保护好耕地。加强环境监测预警和应急能力建设，构建环境与生态安全保障体系。发展低碳产业，倡导低碳消费方式。积极开展生态文明试点创建，全面推进环境保护模范城市和生态市建设工作，把我市建设成为经济高效、环境优美、人与自然和谐统一的生态文明城市。

十、强化科技教育创新，建设聚才扬帆之城

坚持实施科教兴市和人才强市战略，强化科教创新和人才队伍建设，为我市经济社会发展提供强有力的科技和人才支撑。坚持教育优先地位，加大教育投入，基本普及学前教育和高中教育，重点发展高等教育，大力发展职业教育，积极发展民族教育，关心支持特殊教育。2015年，新增劳动力平均受教育年限达到13年左右，人口整体素质较大幅度提高。积极建设创新型城市，增强自主创新能力，建立以企业为主体、市场为导向、产学研相结合的技术创新体系。构建科技创新服务平台，加快企业孵化基地和开发基地的培育和建设，健全创新激励机制，抓好质量兴市工作。加强人才资源开发和能力建设，实施“聚才扬帆”等人才开发重点工程，培养、引进一批专门优秀人才、急需的高层次人才和一大批高素质劳动者，2015年全市人才总量达12万人左右。

十一、发展社会民生事业，建设幸福和谐防城港

强化政府公共服务职能，逐步建成布局合理、功能完善的基本公共服务体系。深化医药卫生体制改革，提高基本医疗卫生服务能力。完善城乡医疗救助制度，加快三级医疗卫生服务体系建设，健全覆盖城乡的基本医疗保障制度。建成三所“三甲”医院。推进文化体育事业发展，打造面向东盟的文化交流基地和滨海运动休闲之城。抓好征地搬迁安置工作，解决被征地农民后顾之忧。做好兴边富民和扶贫工作。加强就业服务，着力提高城乡居民收入水平，健全城乡社会保障体系。抓好计划生育与人口工作，积极发展妇女儿童、残疾人事业。加强国防动员和后备力量建设。创新社会管理，完善社会矛盾调处机制，强化安全生产和安全管理，健全社会应急管理体系，增强防灾减灾能力，加强民主法制和精神文明建设，创建社会和谐稳定模范城市。

2011年工作安排

2011年是“十二五”开局之年。做好今年的工作，对于巩固和发展“十一五”取得的成就，顺利完成“十二五”各项任务，具有重大意义。

今年我市经济社会发展的主要目标是：地区生产总值增长17%以上；财政收入增长17%以上；全社会固定资产投资增长30%以上；全部工业总产值增长25%以上；社会消费品零售总额增长18.6%以上；外贸进出口额（海关）增长15%以上；实际利用外资增长20%以上；引进内资增长10%以上；港口货物吞吐量增长11.8%以上；城镇居民人均可支配收入增长10%以上，农村居民人均纯收入增长10%以上，城镇新增就业人数2.2万人以上，城镇登记失业率控制在3.8%以内；人口自然增长率控制在10‰以内；万元地区生产总值能耗、二氧化碳排放以及化学需氧量、氨氮、二氧化硫、氮氧化物等主要污染物排放总量控制在自治区要求范围内。

为实现上述目标，按照市委统一部署，以“项目建设提速年”、“开放突破年”、“全民创业年”、“民生改善年”为主题，以“建特区、上产业、造新城、强大港、统城乡、旺旅游、兴文化、促民生”为主攻方向，突出抓好以下工作：

一、实施“项目建设提速年”，掀起投资建设新高潮

加快项目建设，力争完成全社会固定资产投资480亿元以上，实施超千万元项目200个以上。

全力推进“天字号工程”建设。核电项目完成投资45亿元，建成220千伏变电站，争取国家批准3～6号机组开展项目前期工作。钢铁项目完成投资10亿元，推进20万吨码头及办公大楼、生活基地等配套项目建设，争取主体工程开工。铜镍项目完成投资35亿元，加快落实项目用海、配套项目和办公商住用地，完成雷达站等迁建工作，开工建设40万吨铜冶炼项目，确保 2012年投产。

继续实施“双百工程”。产业发展“百项工程”计划实施项目206个，总投资1500亿元，年度完成投资160亿元。城市建设“百项工程”计划实施项目228个，总投资610亿元，年度完成投资260亿元。

加快重大基础设施建设。开工防城至江山一级公路，续建防东高速公路、玉罗岭至李子潭一级公路等项目；基本建成沿海铁路扩能改造工程防城港段、钦崇高速公路上思段等项目。加快上思至企沙一级公路等项目的前期工作。建成农村道路103公里。继续完善六大产业园区路网、供水供电、污水处理等基础设施。

二、以建设东兴试验区为引领，实现开放合作新突破

抓住东兴重点开发开放试验区纳入国家战略的重大历史机遇，实施“开放突破年”，推动开发开放再上新台阶。

启动东兴试验区建设。成立试验区管委会，统筹各方力量，高效有序推进相关工作。完成试验区实施方案的制定和报批；启动试验区各项规划。组织一批项目招商。开工建设中越北仑河国际商贸城、国际物流园区等一批项目，尽快完成国门广场改造等重大工程，加快推进防东高速铁路等项目前期工作。在互市贸易、通关便利化等方面积极先行先试。

加大招商引资力度。以中国—东盟博览会、中越边境（东兴—芒街）商贸旅游博览会等重大会展活动为平台，落实工作责任，采取产业招商、园区招商、专业小分队招商等方式，重点开展第三产业、临港工业、战略性新兴产业等方面的招商引资工作，加强历年签约项目的跟踪落实，力争年内引进关联项目投资额180亿元以上。

不断扩大对外贸易。充分利用自贸区零关税政策，扩大与东盟对接，开展资源性互补贸易，拓宽进出口渠道，抓好粮油、豆粕、机电、化工、煤炭、矿石和先进技术设备等大宗商品进出口。扶持和鼓励发展一般贸易、加工贸易、服务贸易，做大做强边民互市等边境贸易。

三、加快“造新城”步伐，不断完善城市功能

以“造新城”为主攻方向，继续抓好“四沿线、四片区”建设，不断完善城市功能，提升城市公共服务能力。

加快推进新城建设。重点抓好“四沿线、四片区”道路交通等基础设施和公交、学校、农贸市场等服务设施建设，不断拓展新城区城市功能。“四沿线”要全面完成北部湾大道建设，加快建设沿线建筑和景观。“四片区”重点是加快行政中心片区开发建设，文化艺术中心、青少年活动中心、科技图书馆、博物馆四大场馆建成使用，金海湾等一批商住房交付使用。中央商务片区的CBD项目开工建设100万平方米以上，尽早完成主楼设计实现开工。中心区北部片区要加快路网工程和文苑公寓等房地产项目建设，启动防城江红头坝大桥和东岸沿江道路及景观建设。桃花湾北部片区要加快完善路网，重点推进台湾城、东盟外滩、北环经济适用房、惠民公共租赁住房等项目建设。西湾新城要加快西湾景观大道、“梦幻北部湾”演出场馆、核电办公及生活基地、金川生活区等项目建设。

加快城市休闲景观建设。加快推进仙人山公园二期、白鹭公园、金花茶公园、伏波文化广场等公共设施建设，规划建设沙潭江湿地公园。加快西湾海域整治、西湾景观照明、桃花湖公园等城市景观工程建设，不断提升城市品位。

加快旧城与城乡风貌改造。加快实施北部湾商业中心、腾飞广场等旧城改造项目，富裕路世界风情步行街建成使用；继续实施小街小巷改造工程，推进渔洲城工业园的整体搬迁，调整河西新区功能和布局。继续推进一批交通要道沿线的城乡风貌改造。

加快提升城市管理水平。建立“两级政府、三级管理”的城区管理体制，逐步实现城市管理规范化、制度化。加大市容市貌、环境卫生专项整治力度，完善市政设施，实施城市绿化、美化、亮化工程，改善人居环境。加强城市交通综合整治，增开公交线路，改善城市交通环境。提高市民素质和城市文明程度。

四、加快兴产业调结构，做大做强工业经济

坚持“以工强市”，加快培育壮大产业集群和园区经济，以更多经济增长点支撑工业经济发展。

培育壮大临港产业集群。加快发展装备制造、冶金、磷酸深加工、粮油食品加工等优势产业，延长产业链，提高产业附加值。建成汇通物流石化仓储、枫叶菜籽油等项目。做好工业挖潜，支持惠禹饲料、中一重工、上思华润水泥等企业扩大生产线。加快金属镍及镍合金系列产品、粮食综合加工等项目技改。

大力发展园区经济。加快园区产业集聚，力争园区产值超400亿元，其中粮油食品产业园要实现产值超200亿元，大西南临港工业园力争产值突破150亿元。完成企沙工业区控制性详细规划，启动国家级企沙经济技术开发区申报工作。

加快发展资源型加工业。充分发挥海洋、农林和进口资源等优势，大力发展制糖、林产林化、建材、橡胶、红木、海产品等资源型加工业，抓好上思华林二期、怡诚食品等一批加工项目。

五、发展大商贸大旅游，推动第三产业大提升

大力发展以商贸旅游为重点的第三产业，聚人气旺市场，繁荣城市经济。

着力壮大商贸物流。加快建设行政中心片区、桃花湾片区等重点区域的商业特色街区，推进建材家居、五金机电等一批专业市场建设。加快推进防城港保税物流园区、中外运防城港仓储物流基地、冲仑物流园等综合物流园区及一批专业物流配送中心建设。扶持物流企业发展，支持开展汽车信贷业务，鼓励外挂车辆回流。扶持发展航运业，引进培育一批航运企业。

着力发展文化旅游。继续实施旅游及文化基础设施和景区景点建设大会战，加快江山半岛旅游区、南山旅游区等十大重点旅游区建设，推进白浪滩、红石谷等景区创A工作。打造红沙群岛等一批特色景点景区。扎实推进“梦幻北部湾”项目，确保五月首演。挖掘文化内涵，提升海上国际龙舟节、京族哈节、森林旅游节、金花茶节等节庆水平。突出高山漂流特色，打响“中国漂流之乡”品牌。建成市游客接待中心及德城等五星级酒店，提升旅游接待能力和服务水平。组织开展好边境游异地办证业务，引进一批有实力的旅行社，积极拓展跨国旅游市场。推出上思十万大山—东兴沿边公路自驾游、“三岛三湾”游等精品旅游线路，运营好防城港至下龙湾海上航线，形成更多旅游新亮点。

着力提升房地产品质。引导房地产持续健康发展。引进知名企业开发一批高档商住小区，打造海景房品牌。新建商品房建筑面积200万平方米以上。深入开展城市规划建设和旅游大篷车宣传促销活动，吸引更多外地人到我市观光旅游、投资置业。

着力培育现代服务业。举办一批重大会展及论坛，繁荣会展经济。出台优惠政策，努力打造“总部经济”。引进和发展一批层次高、带动强、辐射广的金融、信息、规划设计、会计事务、物业管理等附加值高的服务业。

六、着力强基础旺物流，加快建成亿吨大港

坚持“以港兴市”战略，加强港航基础设施建设，加快港口物流发展，确保全市港口货物吞吐能力突破1亿吨。

抓好港航基础设施建设。抓紧修编《防城港总体规划》，加强岸线保护与管理。建成18～22号泊位、防城港20万吨级航道，加快东湾403～407号泊位、钢铁基地20万吨级码头、东湾513～516号泊位等项目建设。推进40万吨级干散货码头、30万吨级码头及航道等项目前期工作。

做大做强港口物流。大力拓展“水转水”业务，积极开辟新航线航班，完善集装箱多式联运系统，优化物流链，降低综合物流成本，吸引更多货物通过防城港中转。建成第二铁路调车场，实现整列发运。落实扶持港口发展的政策措施，破解集疏运、货源组织、航线开辟等方面的难题，进一步强化港口服务和运营管理。

七、努力搭平台激活力，掀起全民创业热潮

实施“全民创业年”，进一步优化创业环境，壮大民营经济，发展微型企业，促进全民创业。

优化创业环境。建立创业指导服务中心，强化信息引导，开辟创业行政审批“绿色通道”。加强创业扶持基地建设，鼓励和引导大中专毕业生、科技人员、复转军人、下岗职工自主创业。

壮大民营经济。加大对民营经济的资金、信贷、土地、税收支持，为企业提供“VIP”服务。最大限度取消民间投资限制，支持各类创业主体围绕我市特色资源、优势产业发展配套加工。继续开展十强民营企业和个体工商户评选表彰活动。

发展微型企业。做好发展微型企业的先行试点工作，设立专项资金扶持一批微型企业发展。年内新增微型企业150家，新增就业人数2000人。

八、突出特色抓“三农”，增强县域经济实力

按照城乡统筹的要求，着力发展特色农业，进一步夯实“三农”基础，壮大县域经济实力，加快城乡一体化进程。

突出特色抓“三农”。按照“稳粮保供给、增收惠民生、改革促统筹、强基增后劲”的基本思路，毫不松懈地抓好“三农”工作。稳定粮食生产，主攻“菜篮子工程”，大力发展“红金”系列特色农业，加快建设糖料蔗、热带水果、畜牧水产、经济林材和无公害蔬菜等生产基地，发展农副产品精深加工，培育壮大一批龙头企业。以“六统强基”为抓手，建设一批新农村示范村。实施“名镇名村工程”，重点扶持有产业支撑、有文化底蕴、群众积极性高的行政村发展休闲观光农业。加强农业基础设施建设，新建或修复一批农田水利基础设施。完善农业服务体系。年内完成3万人次的农民培训任务，转移农村劳动力2万人。

增强县域经济实力。加大对县域发展的指导和扶持力度，加快推进城乡一体化建设，实现城乡协调发展。港口区要继续服务好重大项目建设，壮大园区经济。防城区要更加注重发展城市经济，调整优化产业布局，建设重大项目后勤保障基地。上思县继续发挥资源优势，发展资源型加工业，加快融入沿海沿边经济圈。东兴市要全力推进试验区建设，在开发开放领域先行先试。

九、积极拓财源优结构，强化财税金融保障

加强财源建设。建立多元高效、后劲十足的财源体系，确保支柱财源稳定增长。加强综合治税，提高税收征管水平，优化财源结构。积极争取上级更多资金支持。

优化支出结构。继续加大教育、医疗卫生等民生社会事业投入，确保重点支出。严格投资评审，加强工程审计，提高资金使用效率。强化债务管理，建立风险评价监测制度，防范和化解财政风险。

强化金融保障。提升银政企合作水平，鼓励更多金融机构入驻我市，进一步完善金融组织体系。创新项目建设融资方式，促进以政府性投资为主向社会性投资为主转变，以多元融资加快项目建设。积极引导符合条件的企业上市融资。

十、继续实施“民生改善年”，全面发展社会事业

始终把民生工作作为天大的事来抓，深入实施“民生改善年”，切实解决好群众最关心、最直接、最现实的利益问题。

全面推进民生项目。继续加大对民生项目的投入，年内实施民生项目93个，计划投资17.7亿元。规划建设一批搬迁群众安置区，加快安置区配套设施建设。抓好华侨农林场改革和发展。建成一批保障性住房和公益福利项目。新建市精神病院、上思光荣院等项目。实施母婴健康幸福工程。

加强就业和社会保障。扩大就业再就业，重点抓好高校毕业生、城镇就业困难人员、被征地农民、农民工等四类就业困难群体的就业工作，实现新增就业2.2万人，全面动态消除“零就业”家庭。扩大养老、医疗、失业等各项社会保险覆盖面。实现新农保试点地区居民参保率达80%以上，确保2.6万农村老年人享受农保待遇。进一步完善和规范孤儿救助管理工作，巩固孤儿养育经费标准自然增长机制。

全面发展各项社会事业。认真实施第五轮创新计划，继续深化科技“厅市会商制度”。扎实推进2010—2012年教育发展重点工程和改革试点，逐步实行义务教育以城区为主，高中、中职和高等教育以市为主的教育管理体制；合理调整学校布局，建成职教中心、桃源路小学、北港小学，确保今年秋季学期招生；深入开展职业教育攻坚战，确保通过自治区检查验收。大力引进和用好各类专业人才。加大全民健身工程、农家书屋等惠民项目的建设力度。认真组织备战自治区第十二届运动会。继续实施农村公益电影放映工程。深化医药卫生体制改革，完成市第一人民医院、市中医院创“三甲”工作，健全以县级医院为龙头、乡镇卫生院和村卫生室为基础的三级农村医疗卫生服务网络；抓好疫病防控工作；确保新农合参合率达到95%以上。抓好人口计生工作，继续开展县（市、区）创建国家计划生育优质服务单位。依法规范土地管理，加强耕地保护，合理开发利用土地。加强价格调控监管，确保居民消费价格总水平控制在自治区目标范围内。抓好“质量兴市”工作。做好统计和发展研究工作，为经济社会发展提供科学决策咨询。进一步加强精神文明建设。老龄、工会、青年、妇女、儿童、残疾人、气象、档案、修志、新闻出版、机关事务、防震减灾等各项工作都要取得新的成绩。

加强社会维稳和双拥工作。建立健全突发性事件应急处置机制，妥善处理人民内部矛盾，依法打击走私、贩毒等各种违法犯罪活动，防范和整治各种突出的社会治安问题。加强安全生产工作，坚决防范和杜绝特大安全事故的发生。抓好“双拥”和国防动员工作，加强边海防管理和建设，维护边疆民族地区安全稳定。

努力为民办好10件实事。结合我市实际，集中财力办好一批事关民生的实事：

1、建设6个“菜篮子工程”基地。

2、新建200个村卫生室。

3、建设74个村级公共服务中心。

4、规划建设市中心幼儿园1所，乡镇中心幼儿园4所。

5、在桃花湾广场建设儿童游乐园。

6、实施朱砂港、仙人湾、西茶等城中村改造。

7、在城区新建垃圾中转站6个、公厕6个，新增公共停车泊位3000个。

8、新建廉租房948套、经济适用房629套、公共租赁住房200套、限价房72套。

9、实施贫困残疾人危房改造750户。

10、新建市流浪未成年人保护中心和儿童福利院。

加强政府自身建设

完成“十二五”各项工作任务，必须加快转变政府职能，提高行政效能，进一步优化政务环境。

强化执行，提升行政效能。全面贯彻《关于加强科学民主决策提高行政执行力的意见》等文件精神，提升政府各部门执行市委、市政府决策的能力和水平，切实解决影响政府执行力的突出问题。按照精简、统一、效能的原则，深化政府机构改革。

强化服务，优化发展环境。深入开展审批大提速、机制大健全和责任大落实行动，完善各级政务服务中心。加快事业单位改革步伐，完善公共服务体系。加强电子政务建设，整合政府网站资源，明确部门职责，及时更新、公开各项政策等政府信息，及时办理回复群众意见。

强化督查，完善制度建设。继续开展“百人评议机关”活动，强化督查督办和行政问责，完善行政效能监察和绩效考评制度，确保责任落实和目标完成。

强化依法行政，加强政风建设。抓好“六五”普法，提高依法行政的意识和能力。强化行政执法队伍建设，规范行政执法行为。完善监督机制，自觉接受人大、政协监督，广泛听取各方意见。认真办好人大代表建议和政协委员提案。充分发挥工会、共青团、妇联等群团组织的作用。巩固和扩大基层民主。抓好廉政建设，努力打造廉洁、务实、高效的服务型法治政府。

各位代表！

春潮涌动北部湾，千帆尽举防城港。我们正处在新的历史时期，站在新的发展起点上，新的目标令人鼓舞，新的形势催人奋进。让我们在市委的坚强领导下，继续只争朝夕、废寝忘食地干，“白加黑”、“五加二”地干，“人一之我十之、人十之我百之”地干，同心发力，抓早抓准，抓紧抓实，再创“防城港速度”，再造“防城港模式”，为实现“十二五”规划和“富民强市、幸福和谐”的宏伟目标而努力奋斗！
